# ‘It's been adapted rather than impacted’: A qualitative evaluation of the impact of Covid‐19 restrictions on the positive behavioural support of people with an intellectual disability and/or autism

**DOI:** 10.1111/jar.12859

**Published:** 2021-01-22

**Authors:** Karen McKenzie, George C. Murray, Rachel Martin

**Affiliations:** ^1^ Department of Psychology Northumbria University Newcastle Upon Tyne UK; ^2^ NHS Lothian Edinburgh UK

**Keywords:** behaviours that challenge, covid‐19, intellectual disability, positive behavioural support, qualitative, quality of life

## Abstract

**Background:**

We used a qualitative approach to explore the experiences of social care staff regarding the provision of positive behavioural support (PBS) to people with an intellectual disability at the height of the Covid‐19 restrictions.

**Method:**

We conducted semi‐structured interviews with 19 staff who had recently completed a PBS workforce development programme. Data were analysed using thematic analysis.

**Results:**

Three themes were identified in the context of the restrictions: The challenges to maintaining quality of life and PBS of the people being supported and staff attempts to overcome these; the ways in which PBS and behaviour support plans were implemented and the impact on behaviours that challenge; the ways in which PBS principles were applied at organisational levels to help to understand and address staff stress and distress.

**Conclusions:**

Overall, the staff identified many unexpected benefits of the restrictions. The results are discussed in the context of the study limitations.

## 1 INTRODUCTION

‘Lockdown’ restrictions were introduced across the United Kingdom (UK) as a response to the rapid spread of the Covid‐19 coronavirus. On 16th March 2020, the Health Secretary announced the requirement for all unnecessary social contact to end. These restrictions were formalised in the Health Protection (Coronavirus, Restrictions) (England) Regulations (UK Government, 2020), which came into force on the 26th March 2020. All but essential businesses and activities were closed, households had to significantly restrict their contact with others, and most people could only leave their home for essential activities, such as work. Meetings between more than two people in public places were banned and people were only able to exercise outside once a day. Vulnerable people were also asked to ‘shield’ by staying at home (UK Government, 2020).

Research suggests that people with an intellectual disability are more vulnerable to Covid‐19 and its associated restrictions. A review by Public Health England (2020), conducted between February and June 2020, indicated that the estimated Covid‐19 death rate for people with an intellectual disability was between 3.1 and 3.6 times that for the general population, when taking account of under‐reporting and depending on the source of the data.

This may be because people with an intellectual disability are more likely to have existing health conditions that may place them at increased risk (Emerson et al., 2016), such as respiratory disease (Turk et al., 2020); they are more likely to live in settings with larger groups of people (Landes et al., 2020); and they are more likely to require physical contact to help them with activities such as personal care (Boyle et al., 2020).

A number of authors have also reported concerns that the Covid‐19 restrictions are likely to be particularly detrimental for people with an intellectual disability (e.g. Courtenay & Perera, 2020; Grier et al., 2020; Johnson et al., 2020). By definition, people with an intellectual disability have difficulty with their cognitive and daily living skills (American Psychiatric Association, 2013) and as a result, frequently rely on others to access activities and maintain relationships (Courtenay, 2020). The digital divide (McKenzie, 2007) means that many people with an intellectual disability may be unfamiliar with the use of online technology as a means of making and sustaining contact with others, a mode of communication relied on much more during ‘lockdown’ (Zaagsma et al., 2020). Many rely on routine, structure and predictability to help them to make sense of and navigate their daily lives. In addition, they may have difficulty fully understanding the reasons for the restrictions that have been placed on their lives and disrupted their routines (Courtenay & Perera, 2020).

It has been suggested that the restrictions are likely to cause increased confusion and distress for many people with an intellectual disability (Grier et al., 2020). There has also been some research indicating the negative impact on the mental health, forward planning and coordination tasks of social care staff working under these circumstances (Embregts et al., 2020). In combination, this may result in increases in behaviours that challenge (Courtenay & Perera, 2020). The research into this is limited, although Schuengel et al. (2020) did report an increase in incidents reports of aggression in the Netherlands, as the restrictions there continued. Behaviours that challenge are relatively common in people with an intellectual disability, with recent figures suggesting that they are displayed by about 18% of people who are known to services (Bowring et al., 2019) and are considered to serve a function for the person in expressing an unmet need (Gore et al., 2013).

Positive behavioural support (PBS) is increasingly being used as a way to reduce the need for people to display behaviours that challenge by helping to ensure that the person has individualised support that promotes quality of life through meaningful activities and relationships, reflects their preferences and enhances their skills (Gore et al., 2013). Active support, which provides targeted support, based on an assessment of the individual's strengths and needs is seen as one way of achieving this (e.g. Ockenden et al., 2014). PBS also uses behaviour analysis to assess and identify the function of behaviours that challenge when they do occur. This information is used to develop proactive approaches that aim to reduce the occurrence of the behaviour, including helping the person develop more adaptive functionally equivalent behaviours, and reactive approaches to maintain safety when behaviours that challenge do occur (Gore et al., 2013).

A recent workforce development approach in the North East of England included the free provision of accredited university programmes to social care staff across the region. This programme was developed and delivered in conjunction with colleagues in the NHS, following a region wide scoping exercise. This highlighted the need for a practice‐based approach to workforce development that was standardised, accredited, provided transferable qualifications and competencies that were relevant to the particular role of the individual (see McKenzie, McNall, et al., 2020 for details).

The programme provided online and face to face teaching, combined with workplace coaching, support and supervision (see McKenzie, Martin, et al., 2020 for details). The approach was delivered based on a tiered cascade model, with organisational leads being supported by the programme staff to provide support and supervision to PBS facilitators within their organisation, who in turn provided this to front line staff. As part of the evaluation of the approach, data were gathered prior to staff commencing the programme, immediately after, and three months after they completed it. The last time point coincided with the Covid‐19 restrictions. This provided the opportunity to explore in some detail the experiences of the staff when trying to provide PBS in the context of these restrictions. The study addressed the question: ‘what is the impact of the Covid‐19 restrictions on the provision of PBS in social care settings to people with an intellectual disability?’.

## 2 METHODS

### 2.1 Design and ethics

The study adopted a qualitative approach, using a purposive sample of staff who had completed the PBS programme. Data were gathered via semi‐structured phone interviews, conducted between 3rd April and 28th May 2020. The study context was an exploration of the impact of the Covid‐19 restrictions on staff in the North East of England, who had attended the PBS programme, and their ability to provide PBS. Ethical approval for the study was granted by the first author's university ethics panel.

### 2.2 Participants

The study had 19 participants who represented 19 organisations. Participants were included if they were adults, had completed the PBS programme, consented to take part and worked in a social care setting supporting people with an intellectual disability and/or autism. Four participants were male and 15 were female. Ages ranged between 19 and 60 and all were white British (see Table 1 for further details). Participants had one of the following PBS qualifications: Postgraduate Certificate/Advanced Diploma in Leading PBS, Certificate in Facilitating PBS in teams, or Award of competence in PBS. The first was undertaken by organisational leads/senior staff, the second by staff such as team leaders, who both offered direct support and led teams within services, the third by those whose role was primarily to provide direct support. The first and second awards were based on the successful completion of three modules, and the third award was based on completing two modules. Each module lasted for 3 months and covered topics such as functional based assessment, skills teaching and active support (see McKenzie, Martin, et al., 2020 for further details).

**TABLE 1:** Information about participants

Participant	Age	Gender	Role	Nature of organisation
1	49	F	Registered manager/director	Local
2	45	F	Bank staff nurse	Local
3	19	F	Customer care co‐ordinator	Local
4	37	F	Team leader	Local
5	45	F	National practice development lead	National
6	39	F	Head of PBS practice design	National
7	57	M	Regional PBS lead	National
8	43	F	Area manager	Local
9	52	F	Senior care support	Local
10	50	F	Day service manager	Local
11	28	F	Therapeutic services manager	Local
12	55	F	Staff development manager	Local
13	52	F	Psychologist	National
14	39	F	Head of service	National
15	60	M	Support worker	Local
16	41	M	Team manager	Regional
17	55	F	Area manager	National
18	30	M	Registered manager	Local
19	41	F	Performance coach	National

### 2.3 Procedure

Participants were recruited via existing contact details and were chosen to represent a mix of different organisations (local, regional and national) and different roles within the organisations. They were initially contacted by phone and provided with information about the study. If they consented to take part, a suitable time was arranged to conduct the phone interview.

An interview schedule was used to ensure consistency in the key areas that were covered, while still enabling themes that were relevant to particular participants to be explored. This was designed to address areas that are key components of PBS (e.g. Gore et al., 2013) and which had been explored in the wider evaluation study (McKenzie, Martin, et al., 2020). It included questions about the impact of Covid‐19 on the quality of life, activities, behaviours that challenge and relationships of the people with an intellectual disability who were being supported by the participants. In addition, participants were asked about the impact on staff stress, their ability to implement PBS and behaviour support plans in practice, and the role (if any) of PBS in helping them cope with the negative impact of the restrictions. These interviews were recorded, subsequently transcribed and analysed as outlined below.

### 2.4 Data analysis

Data were analysed using thematic analysis (Braun & Clarke, 2006). This approach was chosen because it is flexible and enables insights into the subjective perspectives of multiple participants (Nowell et al., 2017). It does not require the researcher to hold a specific theoretical position, although Braun and Clarke (2020) note that this does not mean that it exists in a ‘theoretical vacuum’ (p18). The research team adopted a constructivist approach. The first and second authors (who undertook the data analysis) were both experienced researchers and clinical psychologists who have worked with people with an intellectual disability for over 30 years. The interviews were conducted by an experienced research assistant/psychology graduate. All authors had worked on the wider PBS evaluation project but were not involved in the design or delivery of the PBS programme.

The guidance provided by Braun and Clarke (2006, 2020) was followed during the process of data analysis. The individual transcripts were read in detail and considered as a single set of data. Sections from each interview were combined under initial codes as a first to developing relevant themes. Any potentially identifying information was removed or altered at this stage. These initial codes were then combined to create themes and associated subthemes relevant across all interviews. These were shared with the research assistant, as the person who had conducted the interviews, to ensure that they were coherent, consistent and relevant. Examples of quotes were then chosen to illustrate the themes and subthemes.

A summary of the results was shared with participants, and they were invited to provide feedback. This was to ensure that our interpretation of the information provided in the interviews reflected their own perspectives and experiences. We did not receive any requests to amend the results or any feedback indicating that they were invalid.

## 3 RESULTS

The analysis identified three main themes and associated subthemes, as summarised in Table 2.

**TABLE 2:** Themes and associated subthemes

Theme	Description	Associated subthemes
‘It's been adapted rather than impacted’	Explores the barriers that the Covid−19 restrictions placed on the routines and activities that had previously contributed to the quality of life of those being supported and the ways in which staff tried to address these	‘Structure, routine and predictability’
		‘We can't recreate family’
‘Everything's the same but it's not quite the same’	Explores the ways in which staff attempted to sustain PBS and implement behaviour support plans in the face of the restrictions and how this impacted on behaviours that challenge.	‘Stayed pretty even’
		‘There's some learning in that’
‘In practice, it's simple. In theory, it sounds like we've changed the world’	Explores the ways in which PBS principles were applied at wider organisational levels to support staff.	‘We're asking people to risk their health’
		‘There's a presence everywhere’

### 3.1 Theme 1: ‘It's been adapted rather than impacted’

The first theme explored some of the challenges that staff faced in maintaining the quality of life and PBS of the people they supported and their attempts to overcome these.

#### 3.1.1 Subtheme 1: Structure, routine and predictability

Many participants highlighted the importance of maintaining the routines and structure of the people they supported and described the ways in which these were compromised. The Covid‐19 restrictions particularly impacted on external, community‐based activities. The staff attempted to sustain meaningful activities by replicating them within the home: ‘It's been adapted rather than impacted… say a person has an art session or a cookery session, that's normally out in the community, they've created that structure at home’ (P7). There were many attempts to use the new circumstances in positive ways to teach new skills or consolidate existing ones, particularly around hygiene: ‘It actually encourages people to start being more focussed on their own personal hygiene and their own health’ (P11).

There was also an increased focus on active support as a complementary approach to PBS: ‘On the majority of levels, it's been a positive impact, I suppose more so in respect to active support which obviously leads into the PBS’ (P7), and turning what might have been previously seen as mundane tasks into more meaningful activities: ‘We've been trying to do active support, even if it's just getting someone to just undue a button on a coat or take a coat off’ (P15).

#### 3.1.2 Subtheme 2: ‘We can't recreate family’

The importance of social relationships for the quality of life of the people being supported was also recognised and staff explored ways in which these could be maintained as much as possible: ‘We've done a lot around facilitating contact with families via other methods’ (P13). Staff worked hard to achieve these social interactions using technological solutions: ‘So we are looking at different ways we can get family involved…now that we have Skype and we have clients who've bought iPads and things and tablets’ (P14). There was a recognition, however, that this was only a partial substitute for face to face contact and physical affection: ‘We can't recreate family in that sense if people are missing their loved one and getting hugs from mum and dad, we can create other situations that will give them something like that hopefully, give them that warm feeling, set up Skype calls, telephone calls’ (P17).

The restrictions increased the time that staff and those they supported spent with each other. This increased contact was often described as creating stronger relationships between people: ‘There's definitely quite a few people that I'm aware of who seem to be engaging more meaningfully with staff, with peers who they live with…and it's leading to more positive outcomes for them’ (P11). It provided an opportunity for some people to learn to feel comfortable with and trust a new staff team: ‘She was…not getting to know the staff and being quite wary of us…her relationship now with the staff has massively increased because of course she's had to spend time with the staff’ (P13).

The restrictions also created a sense of belongingness, resulting in people sharing more activities, such as taking their meals together: ‘There's a bit more of a communal aspect and not an enforced one in the sense that we're making people eat together… it's a coming together for people in a natural way’ (P17).

### 3.2 Theme 2: ‘Everything's the same but it's not quite the same’

This theme explored how Covid‐19 restrictions affected the ways in which PBS and behaviour support plans were implemented and the behaviours that challenge of some of those people who were being supported.

#### 3.2.1 Subtheme 1: ‘Stayed pretty even’

For most, the ways in which support was delivered and the associated behaviour support plans were implemented were relatively unchanged: ‘The behaviour support plans are still in place and they've still been implemented’ (P1). The main impact was because of the restricted access to community activities, and the requirements to maintain a social distance. The latter impacted on anything that required physical contact, such as responding to affection: ‘He wants to hold hands. You try to have social distancing without sort of letting him know why’ (P15); providing physical support, for example with personal care: ‘the staff need to be there, obviously near people, to support them’ (P14); and physical intervention. It was recognised that there might be a need for some adaptations in light of this: ‘it's about…changing the support plan to adapt to the situation that we're in’ (P7).

Many services had taken proactive steps and adjusted behaviour support plans in anticipation of the lockdown restrictions. In some cases, this involved withdrawing and replacing activities over a longer period: ‘so instead of suddenly saying “oh you can't go anywhere”, we gradually did it over three weeks before anybody was locked down’ (P1), and planning ahead to ensure that the resources that were needed were in place when full lockdown occurred: ‘…so getting the extra resources for people in the service to be able to do things at home’ (P6).

Perhaps because of the maintenance of PBS and the behaviour support plans, few participants reported increases in behaviours that challenged: ‘…pretty much everything has stayed pretty even’ (P6) and many reported a decrease. This was frequently attributed to the new skills and understanding that staff had learned on the PBS programmes:

It's enabled our staff to…understand that behaviours happen for a reason …it's changed staff's attitudes and beliefs and then from that we've been able to respond differently…which has then reduced the behaviours that challenge (P1)

In addition, the reduction in activities and social interactions had reduced demands on those being supported: ‘there's been less behaviours that challenge based on there's been less pressure put on service users’ (P1) and on staff: ‘Some of the demands have been removed off people, both staff and residents.’ (P5).

Where increases in behaviours that challenge had occurred, this was frequently attributed to the difficulty that the person being supported had in fully understanding why their routines had changed: ‘We're seeing an increased number of behaviours because people just don't understand why things are changing’ (P18). At times, staff avoided providing full explanations for changes in order to reduce the person's stress: ‘Everything's the same but it's not quite the same…he just knows somethings different, but we don't tell him what's different…he'd just sort of say you're gunna die, you're gunna die… it would also get him stressed out’ (P15). In this case, some information was withheld about why so many of the rooms in the service were empty and unstaffed, to avoid the person becoming anxious about the health of the staff member.

Where behaviours that challenge did occur, the staff were able to use their PBS learning to assess and intervene quickly to reduce it:

We're continually monitoring, recording any incidents that do occur so that we can still try and spot any trends and patterns and see what the kind of triggers are and if there is anything that we can do to, to minimise the impact of those (P11)

#### 3.2.2 Subtheme 2: ‘There's some learning in that’

The stripped down nature of many people's lives often allowed the function of a person's behaviour to become more apparent: ‘I think we've been able to pinpoint more behaviours, like the function of the behaviour’ (P3). Activities that people had previously habitually undertaken prior to the restrictions were seen as potentially detrimental and as needing to be managed differently when the restrictions eased: ‘She [before lockdown] goes to see all her friends, yet actually what I think is transpiring is it's probably an absolute trigger centre…She's a lot calmer, so I think there's some learning in that’ (P17). Similarly, the living situations of some people were seen as having contributed to past behaviours that challenge:

She moved into a house with another two ladies, those two ladies have gone home during the lockdown period and…she's a lot more happier so obviously when this is over, we need to look at that situation (P12)

Many participants felt that the restrictions had led to an increased rate of change because the staff were experiencing the results of PBS directly and over a shorter time period: ‘It probably would have taken us months and months to train people in active support and get them to understand the value of engagement, whereas they've done this in a week’ (P19). This also contributed to a more rapid and wider culture change in respect of PBS for some organisations: ‘I think we've managed to do in the last couple of months some culture change that I think would have taken me a year and a half’ (P6), with the changes occurring as a natural consequence of the restrictions: ‘If anything it's pushed staff into more active support than they did before without realising it’ (P5). Overall, the restrictions were seen by many as having had many unexpected benefits:

I think that it's been an opportunity to really think about what's going on and how people are given choice in their quality of life, what other alternatives to normal activities can staff create, so on the majority of levels, it's been a positive impact (P7)

### 3.3 Theme 3: ‘In practice, it's simple. in theory, it sounds like we've changed the world’

As well as using aspects of PBS to help minimise the negative impact of Covid‐19 restrictions on those being supported, there was also evidence of the principles being used at a systems level. This theme explores the ways in which PBS principles were applied at organisational levels to help to understand and address the stress that the staff were experiencing while working under Covid‐19 restrictions.

#### 3.3.1 Subtheme 1: ‘We're asking people to risk their health’

Many participants reported that staff initially experienced high levels of anxiety: ‘I think initially …everybody went into sheer panic.’ (P12). PBS was seen as helping the participants to understand the anxieties of their staff and colleagues and support them, in turn, to understand the perspective of people with an intellectual disability:

It [PBS] gives you an outlook of how somebody who needs that support and it emphasises the importance of where your values needs to be…people having an understanding of the overall impact on someone's life (P18)

There was a recognition of the level of risk that staff were placing themselves under: ‘We're asking people to risk their health and their lives and go into a service’ (P6), and that this led to anxiety: ‘From the staff point of view, we're having lots of issues of people's own sort of worries about their safety, their family's safety’ (P18). While recognising the risk, very few participants directly mentioned concern about catching Covid‐19. When this fear was expressed, it was mainly in the context of potentially passing the virus on to those they supported: ‘the staff were frightened in case they brought something into the homes and one of our service users became poorly’ (P1).

#### 3.3.2 Subtheme 2: ‘There's a presence everywhere’

All the organisations had introduced additional staff support measures. These were frequently presented as being within the context of PBS principles, values and culture: ‘We are doing twice weekly welfare calls with staff and that's kind of all part of that PBS culture of “we're here, we're supporting you, if you do feel scared or you've got any issues, you can come to us at any point”’ (P14). Staff also used their experiences on the PBS programme to directly influence strategic responses to Covid‐19 within their organisation: ‘I'm on our COVID response group…because of that we've planned our organisational strategy to include that [PBS] and it's been something that's been talked about at every single meeting and I think before I did this course, I wouldn't have fought for it as much as I have’ (P6).

The cascade model on which the PBS programme was designed and delivered was also seen as very beneficial in ensuring that all services in the region could draw on support from someone with PBS skills:

We've now got people embedded in the majority of the services who have participated in some way with the programme. There's a presence everywhere which I think has created the consistency in the approach (P11)

The changed circumstances also provided greater opportunities for modelling good practice and work‐based coaching: ‘the team leader…they're actually there seeing them work and able to give advice and, you know, be proactive’ (P12).

The PBS emphasis on functional understanding and proactive approaches was also seen as particularly helpful under the circumstances of the pandemic:

That idea of being more proactive, looking at the environment, looking at the normal antecedents, what we know about that person, and anticipating the kind of antecedents that are bound to come up (P17)

This proactive approach had been operationalised by some organisations through the introduction of supportive measures in anticipation of lockdown: ‘We started a lot of the prework weeks and weeks before lockdown happened, so we made sure there was pastoral support sessions in place’ (P6). There were opportunities for staff to take a break from work: ‘Those staff that we have recognised have been feeling a bit more stress that normal, we've asked if they want to…spend a bit of time out of work’ (P12) and the introduction of a range of staff activities: ‘We've got virtual choirs, we've got a virtual book club… we have weekly Skype calls where we do a bit of fun and games’ (P14). Staff teams also supported each other informally, and many drew on their own PBS learning to help with this: ‘I did a few things off the PBS course like some things around wellbeing and…a values game just to help them focus again about what's important at work’ (P13). Many also used the community of practice developed from the PBS programme to gain support for themselves: ‘We still do our meetings with the rest of the people who did the [programme], which has been fantastic…it's good for like informal supervision as well between you as a team’ (P1).

While participants generally felt supported by their organisations, peers and wider staff teams, some were critical of the support and guidance offered at government level: ‘The guidelines and the protocols change every single day’ (P9). This was felt to lead to inconsistent approaches: ‘We have to interpret that ourselves…another service… was doing a different thing with the same guidance’ (P14). It was also felt that the specific needs of people with an intellectual disability were being overlooked in government guidance: ‘There's not enough recognition in the guidance nationally, actually the impact of how we support people with a learning disability’ (P5).

Some social care staff felt undervalued. This was exemplified by the public clapping regularly to show their appreciation of the NHS, rather than social care staff, at the start of the pandemic: ‘At the beginning…social care was just forgotten. There was all the clapping… the clap for the NHS and how brilliant the NHS was, but I don't think that it was ever recognised that social care and how difficult it was’ (P1). When this was extended to clapping for all carers, there was some cynicism that the appreciation would subsequently be matched by better pay for staff: ‘Now there's people outside clapping for us and when this is all over, we'll go back to just being on minimum wage and forgotten about’ (P16).

## 4 DISCUSSION

The research explored the impact of Covid‐19 restrictions on the ability of staff to provide PBS. While the participants articulated a number of challenges to this, in particular because of changes to the routines, activities and direct contact with friends and family of the people they supported, the overwhelming message was that staff had adapted to ensure that PBS was still provided.

Despite concerns being expressed in the literature that the Covid‐19 restrictions would have a significant negative impact on quality of life and behaviours that challenge (e.g. Courtenay & Perera, 2020), few participants in the present study reported that this was the case. The staff described many ways in which they had attempted to maintain the quality of life of those they supported and used proactive, pre‐planning to ensure that people with an intellectual disability engaged in meaningful activities and had ongoing contact with loved ones.

The latter was often achieved by using online technology. Previous research has indicated a digital divide, with people with an intellectual disability being less likely to use online technology and resources (McKenzie, 2007). The present research suggests that this is not inevitable. Indeed, research in the Netherlands found that people with an intellectual disability increased their use of online systems for support during the Covid‐19 restrictions (Zaagsma et al., 2020).

Few participants reported an increase in behaviours that challenge. Where this did occur, it was often linked to the difficulty that the person had in understanding the reasons for changes in their routines and activities. In some cases, staff were concerned that a full explanation would cause the person distress and used omission, that is, withholding some information, as a way of avoiding this. Omission is an example of a ‘therapeutic untruth’ that is, an untruth used in the best interest of the person. The use of therapeutic untruths has been found to be common in intellectual disability services, with over 96% of social care staff in one study reporting using them (McKenzie et al., 2021). They are, however, frequently used in the absence of formal guidance, raising concerns about whether they are always used in ways that are ethical and effective. This also highlights the need to further explore the use of therapeutic untruths in a PBS context (McKenzie et al., 2021). The development of accessible information about Covid‐19 for use by staff supporting people with an intellectual disability (e.g. Mencap, 2020) may also make it easier for them to explain relevant concepts.

Many participants reported a decrease in behaviours that challenge. This was generally attributed to the staff implementing their PBS knowledge in practice and identifying causes of behaviour that had previously been less apparent—such as the demands of visiting a friend or living with others. In a few cases, the new circumstances brought evidence to light that was not previously available and allowed the participants to review and adapt their current approaches in light of these, both of which are important aspects of PBS (PBS Coalition, 2015). The Covid‐19 restrictions also provided an opportunity to reflect on the preferences and needs of the person being supported. In some cases, it may be that what is thought to provide an engaging, active and socially valid lifestyle, actually places excessive demands on the person and contributes to the expression of behaviours that challenge. To date, very little research has been conducted into the impact of the restrictions on behaviours that challenge. Schuengel et al. (2020) found an initial fall in reports of incidents of aggression at the start of the Covid‐19 restrictions. These subsequently increased but did not exceed the range that was reported in the period prior to the restrictions.

The participants also cited ways in which PBS principles had been applied at organisational level to help understand and address staff stress. This included the proactive, pre‐planned introduction of staff support measures, and trying to ensure that all services had access to someone with PBS knowledge and expertise. The participants appeared to feel valued and supported by their colleagues and organisations, if not by the government and the wider public.

The importance of social care staff and the need for them to have protection and guidance that parallels that provided for health care staff have recently been highlighted (e.g. Grier et al., 2020). Participants in our own study and in that by Embregts et al. (2020) have, however, expressed frustration at government responses, disappointment that social care did not seem to be appreciated as much as health care staff. There was also concern at the limited specific guidance in relation to people with an intellectual disability.

The participants in both studies also reported similarities in terms of the need for creativity to deal with the challenges of the Covid‐19 restrictions, the practical difficulties of being unable to maintain social distance when providing direct support, and the importance of collaboration and connectedness with others. There were, however, also some marked differences. Embregts et al. (2020) found that staff used more individualistic coping strategies, such as focusing on their work. There was less evidence of the formal, organisational staff support strategies that were reported in the current study. Embregts et al. (2020) also found that staff had a focus on day to day support, leading to concerns about the quality of support in the longer term. By contrast, our participants continued to implement PBS and related behaviour support plans, to use proactive approaches, and to reflect on lessons that could be learned and acted upon in the aftermath of the restrictions.

In conclusion, while the staff in our study clearly experienced the stress and challenges of working under Covid‐19 restrictions, the majority had a positive focus and could articulate many unexpected benefits of their new working conditions. They continued to provide PBS and adhere to behaviour support plans, using creative methods to adapt them when required. PBS has been identified as a multi‐component framework that combines many elements to provide an effective, systemic approach (Gore et al., 2013) and many staff articulated, either directly or indirectly, the ways that PBS principles had helped them to cope with the new circumstances, at individual, team and organisational levels.

The study did have limitations. The participants were from organisations in the North East of England and all had recently completed a PBS programme. Research with participants from different geographical locations may have yielded different results. The research team was involved in the wider evaluation of the PBS approach in the area and had had some prior contact with the participants. This may have influenced both the participants’ responses and the researchers’ interpretation of those responses.

The participants worked for organisations that supported people with an intellectual disability and/or autism. There were, however, no themes identified that were specific to people with autism. This may be because the participants mainly supported people with an intellectual disability. Research suggests that people with autism, who do not have an intellectual disability, can also experience difficulties with understanding and dealing with the changes in routines and activities related to the Covid‐19‐related restrictions. This can result in changes in behaviour and increased anxiety in the person and parent/carer, similar to those described in the present study (see Mutluer et al., 2020). They may also, however, face specific challenges that differ from those that were highlighted in the present study. Further research is needed to explore this.

An additional issue is that, because the aim of the study was to explore the subjective views of participants, rather than focus on measurable indices of change, there are many factors that could have influenced these views. The sample size was relatively small and participants held different PBS qualifications and roles within organisations. These ranged from leading an organisation to providing direct support. As a result, they were likely to have had different levels of involvement in writing behaviour support plans and using their PBS knowledge to implement them in practice. This may have influenced their views about the impact of the PBS programme on themselves, their colleagues and the people they supported. It may also be that participating in any form of programme that required time and effort may have influenced participants’ perceptions about its impact on the Covid‐19 restrictions, rather than the results being specific to the PBS programme. Despite these potential issues, the themes were found to be largely consistent across participants and to illustrate the application of PBS principles in practice.

Finally, the interviews spanned the period from shortly after the initial ‘lockdown’ restrictions were introduced in the UK, to 2 months after their introduction. It is possible that participants’ experiences would have been different if they had been interviewed at different points during the restrictions.

## CONFLICT OF INTERESTS

None.
